# An Experimental Study of Radiation Effect on Normal Tissue: Analysis of HIF-1α, VEGF, eIF2, TIA-1, and TSP-1 Expression

**DOI:** 10.4274/Tjh.2012.0142

**Published:** 2013-12-05

**Authors:** Caner Aktaş, Cengiz Kurtman, M. Kemal Özbilgin, İbrahim Tek, Selami Koçak Toprak

**Affiliations:** 1 Bülent Ecevit University School of Medicine, Department of Radiation Oncology, Zonguldak, Turkey; 2 Ankara University School of Medicine, Department of Radiation Oncology, Ankara, Turkey; 3 Celal Bayar University School of Medicine, Department of Histology and Embryology, Manisa, Turkey; 4 Medicana International Hospital, Department of Medical Oncology, Ankara, Turkey; 5 Başkent University School of Medicine, Department of Hematology, Ankara, Turkey

**Keywords:** Angiogenesis, Radiation, Cancer and normal tissue, Vascular endothelium, HIF-1α, VEGF, eIF2, TIA-1, TSP-1

## Abstract

**Objective:**

This study investigated whether or not the stress and hypoxia, which are the effects of radiation on normal vascular endothelium, leading to the release of HIF-1α, VEGF, eIF2, TIA-1, and TSP-1 were related and the possibility of them stimulating angiogenesis.

**Materials and Methods:**

Twenty-four male Swiss Albino mice were separated into 4 groups. The first group was the control group (Group 1), and the second, third, and fourth groups were euthanized after 24 h (Group 2), 48 h (Group 3), and 7 days (Group 4), respectively. A single-fractioned 10 Gy of ionizing radiation was applied to all mice’s pelvic zone with Co-60. Bladders were removed completely from the pelvic region. Immunohistochemistry and light microscopy were used to investigate whether there would be an increase or not in the angiogenesis pathway by using the HIF-1α, VEGF, eIF2, TIA-1, and TSP-1 antibodies.

**Results:**

The HIF-1α antibody showed strong staining in Group 3, while the staining intensity was less in other groups. VEGF showed weak staining in Groups 1 and 4, while moderate staining in Group 2 and strong staining in Group 3 was observed. eIF2 showed strong staining in Groups 1 and 4. Groups 2 and 3 were stained weakly. In the present study, staining with TSP-1 was very strong in the samples belonging to Group 1, while other groups showed very weak staining.

**Conclusion:**

When normal tissue was exposed to radiation, the positively effective factors (HIF-1, VEGF, eIF2, and TIA-1) on the angiogenesis pathway were increased while the negative factor (TSP-1) was decreased. Radiation may initiate physiological angiogenesis in the normal tissue and accelerate healing in the damaged normal tissue.

## Abstract (tr)

**Amaç:**

Çalışmada, radyasyonun normal vasküler endotelde oluşturduğu stres ve hipoksiye bağlı olarak HIF-1α, VEGF, eIF2, TIA-1 ve TSP-1 ekspresyonu ile bunların aralarında ilişki olup olmadığı ve bu ekspresyonlar neticesinde anjiojenezin uyarılabileceği hipotezi araştırılmıştır.

**Gereç ve Yöntemler:**

Bu amaç ile 24 adet erkek Swiss albino fare, kontrol grubu (1. Grup) ve sırasıyla 24 saat (2. Grup), 48 saat (3. Grup) ve 7. Günde (4. Grup) ötanazi uygulanmak üzere 4 gruba ayrıldı. Co-60 cihazı ile pelvis bölgesine tek fraksiyonda 10 Gy iyonize radyasyon uygulandı. İmmünhistokimyasal metod ve ışık mikroskobu yöntemi ile HIF-1α, VEGF, eIF2, TIA-1 ve TSP-1 antikorları kullanılarak mesane damar endotelinde anjiojenez yolunda yer alan faktörlerin ekspresyonları değerlendirildi.

**Bulgular:**

Çalışmamızda iyonize radyasyon ile mesane damar endotelinde HIF-1α antikoru ile 3. grupta kuvvetli boyanma izlenirken diğer grupların boyanma şiddeti daha az idi. VEGF incelendiğinde, 1. ve 4. grupta zayıf boyanma izlenirken, 2. grupta orta derecede, 3.grupta ise kuvvetli boyanma izlendi. eIF2, 1. ve 4. grupta kuvvetli boyanma göstermiş, 2. ve 3. grupta ise zayıf boyanmıştı. TIA-1 ile yapılan değerlendirmede ise 1. grupta zayıf boyanma özellikleri gözlenirken, 2. ve 4. grup orta derecede, 3. grupta ise oldukça kuvvetli boyanma izlendi. Çalışmamızda TSP-1 ile boyanma 1. grup örneklerde oldukça kuvvetli gözlenirken, diğer gruplarda boyanma özellikleri oldukça zayıf izlendi.

**Sonuç:**

Radyasyon uygulaması ile normal dokuda anjiojenez yolundaki pozitif etkili faktörlerin (HIF-1α, VEGF, eIF2, TIA-1) arttığı görülürken, negatif etkili faktörün (TSP-1) düştüğü belirlendi. Bu sonuç ile radyasyona yanıt olarak normal dokuda fizyolojik anjiojenezin başlatılarak doku iyileşmesinin hızlandırılmaya çalışıldığı düşünülmüştür.

## INTRODUCTION

Introduction Treatments with radiotherapy targeting tumor cells and tissues can lead to potentially lethal and sublethal damage in vascular and perivascular structures, normal tissues, and cells. Tumor cells with good vasculature are more sensitive to radiation, while tumoral structures with poor oxygenation are more resistant to radiation [1]. The response of the normal vascular endothelium and tumor vasculature to radiation treatment is important. 

Radiation treatment leads to direct cell death by developing fractures in the double-helical DNA structure, while the development of reactive oxygen species (ROS) is another reason for cell death [2]. ROS and the nuclear factor kappa B and interleukin-8, together with the factors that act on angiogenesis, can start the production of vascular endothelial growth factor (VEGF) in an independent pathway with the hypoxia-inducible factor (HIF) [3,4].

With the ionizing, oxidative, and reductive effects of radiation, factors like HIF, the endogenous mitogenic VEGF, the eukaryotic initiation factor 2 (eIF2) that determines the response of the cell towards stress, and T-cell intracytoplasmic antigen-1 (TIA-1) can be released [5]. Thrombospondin-1 (TSP-1) is the endogenous antiangiogenic factor, which can inhibit these factors that can be released as a response to stress [6]. It is known that factors other than TSP-1 are released during angiogenesis. 

The aim of this study is immunohistochemical determination of VEGF, which can be released with the increase of HIF-1a, being the positive factor that can start physiological angiogenesis as a response to the stress that develops in normal vascular endothelium as a result of radiation and hypoxic states; TIA-1, which determines apoptosis; levels of endogenously released TSP-1, which can inhibit angiogenesis; and eIF2, which is known to determine stress granules. Furthermore, by exposing normal vascular endothelium to ionizing radiation, the relation between negatively influencing TSP-1 and the positive factors HIF-1, VEGF, eIF2, and TIA-1 of angiogenesis pathways is investigated. The present study tries to determine the varying conditions of HIF-1a, VEGF, eIF2, TIA-1, and TSP-1, all of which play a role on angiogenesis signaling pathways, and their interrelations by the immunohistochemical method at 24 h, 48 h, and 7 days. 

## MATERIALS AND METHODS

A total of 24 healthy, male, adult Swiss Albino mice, weighing 30-40 g, were obtained from the Gazi University Experimental Animal Laboratory and used as subjects. The subjects were isolated from stress and noise and fed with water and food ad libitum at 25 °C in a cycle of 12 h of dark and 12 h of light before being included in the study. Care of the subjects was performed at the Gazi University Experimental Animal Laboratory throughout the study. The subjects were divided into 4 groups, the first being the control group and each containing 6 mice. Except for the 6 subjects in the control group, mice were exposed to ionizing radiation at their pelvic region on the same day with the Co-60, 780-C device present in the Department of Radiation Oncology of Gazi University School of Medicine, applied to a region with 5 a 23 cm dimensions with a source-to-surface distance of 80 cm for 10.7 min with a 1000 cGy dose for Dmax in a single fraction. For obtaining sedation before the procedure, intramuscular ketamine injection at a dose of 45-50 mg/kg was performed. In the field, 5 mice were fixed at the prone position.

The 6 mice in Group 1 (control) were not exposed to any radiation. Euthanasia by the method of cardiac puncture blood collection on the first day was performed, following sedation with 45-50 mg/kg intramuscular ketamine injection. After euthanasia, the pelvic region was dissected and the bladder was completely removed. 

The 6 mice in Groups 2, 3, and 4 were sedated with 45-50 mg/kg intramuscular ketamine injection 24, 48, and 168 h after their pelvic region was exposed to a single fraction of 1000 cGy ionizing radiation, respectively, and euthanasia by cardiac puncture blood collection was performed. After euthanasia, the pelvic region was dissected and the bladder was completely removed.

All samples were first washed in a solution containing 10% formol and then placed in screw-cap sampling containers containing 10% formol, with separate boxes used for every animal.

The tissue samples taken after the procedure were embedded into paraffin following the routine light microscopy paraffin tissue method at the Department of Histology and Embryology of Celal Bayar University School of Medicine. Slides of 5 µm were evaluated with the indirect immunohistochemistry method by using HIF-1α (monoclonal antihuman/mouse HIF-1α antibody, cat. no. ab463; Abcam, Cambridge, MA, USA), VEGF (antimouse VEGF antibody; Santa Cruz Biotechnology, Santa Cruz, CA, USA ), TIA-1 (cat. no. ab40693; Abcam), eIF2 (anti-eIF2α antibody, sc-11386; Santa Cruz Biotechnology), and TSP-1 (anti-thrombospondin-1 antibody, cat. no. ab79450; Abcam) primary antibodies.

In the control and experimental groups, the vascular endothelial cells of the bladder tissue were compared with the immunohistochemical method. The effect of ionizing radiation at 24 h, 48 h, and 7 days on HIF-1α, VEGF, eIF2, TIA-1, and TSP-1 levels that play their role in angiogenesis signaling pathways was evaluated and scored under light microscope in accordance with groups for every parameter. 

The intensity of staining was evaluated for every preparation as 1 (weak), 2 (medium), or 3 (strong). The number of stained cells in every field was determined as a percentage and, finally, the histochemical score (H score) of each subject was obtained. 

The study protocol was approved by the local ethics committee for animal experimentation.

**Statistical Analysis**

Analyses of data were performed with SPSS 15.0 for Windows. As the variables used in the study were in accordance with normal distribution, the Shapiro–Wilk test was used, and as the P-values in the test were significant at >0.05, it was concluded that the data were distributed normally. 

During intergroup comparisons of variables, one-way analysis of variance (ANOVA) was used as the number of subgroups exceeded 2. The variance analysis test revealed that the difference between groups was significant. Paired comparisons of groups that were found to be significant with the ANOVA test were performed and the paired comparison of groups with homogeneous variance (equal variance) was made with the Tukey test, while the Tamhane test was used for the comparison of nonhomogeneous groups. The confidence level was accepted as 95% in the performed statistical evaluations. 

## RESULTS

**Figure 1:**
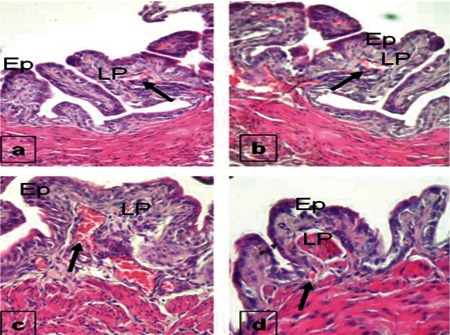
Hematoxylin-eosin staining properties of the bladder in Group 1 (a), Group 2 (b), Group 3 (c), and Group 4 (d). Ep = epithelium, LP = lamina propria

When the hematoxylin-eosin–stained preparations of bladder samples were investigated with the histochemical technique, the bladder lumen in Group 1 (control) was covered with folded and transitional epithelium and the epithelial thickness was in general 5 or 6 layers. The lamina propria under the epithelium consisted of collagen fibers. These collagen fibers were distributed irregularly. Various blood vessels and additional fibroblasts, which are connective tissue cells, were observed within the lamina propria. Under the lamina propria, longitudinal muscle fibers were observed at the inner and outer layers, while circular fibers were seen in the middle. Bladder tissue samples of the experimental groups also revealed transitional epithelium coverage and underlining lamina propria. Slight edema was especially observed in Groups 2 and 3, different from Group 1 (Figure 1). 

**Figure 2:**
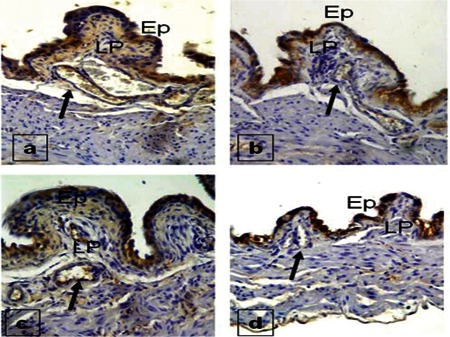
Staining properties of the bladder with VEGF antibody in Group 1 (a), Group 2 (b), Group 3 (c), and Group 4 (d). Ep = epithelium, LP = lamina propria

When the tissue samples were investigated with the histochemical technique, it was seen that Groups 4 and 1 were very weakly stained, while Group 2 showed medium immunoreactivity in preparations stained with the VEGF antibody. VEGF immunoreactivity was shown by a high level of staining in Group 3 (Figure 2). 

Variance homogeneity test results for the VEGF variable showed that the intergroup variance was homogeneous (P=0.099; P>0.05) and the Tukey test was performed. According to the VEGF Tukey honestly significant difference multiple comparison test, there was a statistically significant difference between the control group and Groups 2 and 3 (P = 0.009 and 0.000). However, there was no statistically significant difference between Group 4 and the control group (P = 0.386). Groups 2 and 3 were significantly different from the other groups (P = 0.009, 0.000, and 0.000). Although there was no statistically significant difference between Group 4 and the control group, Group 4 was significantly different from Groups 2 and 3 (P=0.386, 0.000, and 0.000). 

**Figure 3:**
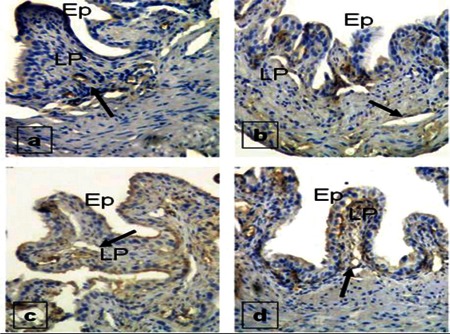
eIF2 staining properties of the bladder in Group 1 (a), Group 2 (b), Group 3 (c), and Group 4 (d). Ep = epithelium, LP = lamina propria.

The eIF2 antibody staining of Groups 1 and 4 revealed very strong staining at vessel walls. In Groups 2 and 3, however, the staining was medium and showed almost similar properties (Figure 3). 

According to the eIF2 Tamhane multiple comparison test, the difference between Group 1 and Groups 2 and 3 was statistically significant (P = 0.000 and 0.001). There was no statistically significant difference between Group 4 and the control group (P = 0.989). Groups 2 and 3 significantly differed from the control group and Group 4 (P=0.002 and 0.001). When Group 4 was examined, the difference from Groups 2 and 3 was statistically significant (P=0.002 and 0.006).

**Figure 4:**
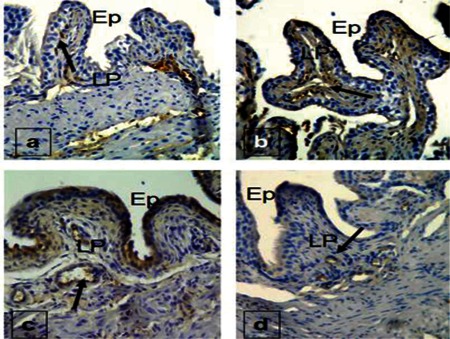
HIF-1 staining properties of the bladder in Group 1 (a), Group 2 (b), Group 3 (c), and Group 4 (d). Ep = epithelium, LP = lamina propria

Staining with the HIF-1α antibody was strong in Group 3, while the degree of staining in other groups was very little (Figure 4). 

According to the HIF-1 Tukey honestly significant difference multiple comparison test, although the control group was statistically significantly different from Group 3 (P=0.000), no statistical difference could be revealed between the control group and Groups 2 and 4 (P=0.253 and 0.960). Group 2 was significantly different from Group 3 (P = 0.005), but no statistically significant difference could be determined for Groups 1 and 4 (P=0.253 and 0.1). Group 3 was statistically different from other groups (P=0.000, 0.005, and 0.000). Group 4 was significantly different from Group 3 (P=0.000). Group 4 was not significantly different from the control group and Group 2 (P=0.960 and 0.1). 

**Figure 5:**
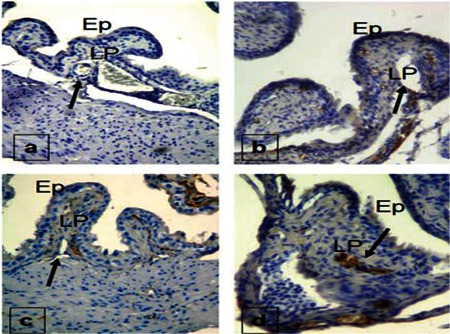
TIA-1 staining properties of the bladder in Group 1 (a), Group 2 (b), Group 3 (c), and Group 4 (d). Ep = epithelium, LP = lamina propria

When tissue samples stained with the TIA-1 antibody were examined, Group 3 showed strong staining while Groups 2 and 4 had medium staining. Samples of the control group were weakly stained (Figure 5).

According to the TIA-1 Tamhane multiple comparison test, the control group was significantly different from Groups 2, 3, and 4 (P=0.000, 0.000, and 0.013). Group 2 was significantly different from Group 3 and the control group (P=0.003 and 0.014). There was no significant difference between Groups 2 and 4 (P=0.809). Group 3 was significantly different from the other groups (P=0.000, 0.014, and 0.000). Group 4 was statistically significantly different from Group 3 and the control group (P=0.013 and 0.000). However, the difference between Groups 4 and 2 did not reach statistical significance (P=0.809). 

**Figure 6:**
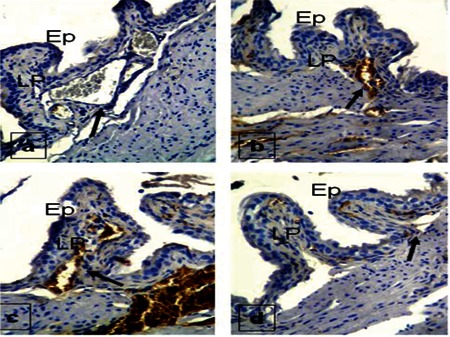
TSP-1 staining properties of the bladder in Group 1 (a), Group 2 (b), Group 3 (c), and Group 4 (d). Ep = epithelium, LP = lamina propria

Staining with TSP-1 was strong in the control group, while staining properties in other groups were seen at low levels (Figure 6). 

According to the TSP-1 Tamhane multiple comparison test, the control group was statistically significantly different from the other groups (P=0.000, 0.000, and 0.000). 

Although Group 2 was statistically significantly different from the control group (P=0.000), no significant difference could be revealed between Groups 3 and 4 (P=0.998 and 0.533). Group 3 was statistically significantly different from the control group (P=0.000). However, Group 3 was not statistically significantly different from Groups 2 and 4 (P=0.998 and 0.169). Although Group 4 was statistically significantly different from the control group (P=0.000), no significant difference could be revealed between Groups 2 and 3 (P=0.553 and 0.169).

The staining properties of all groups are given in Table 1.

## DISCUSSION

The sensitivity of tumor cells to radiation is important in treatment. However, the interest in the radiotherapeutic response for nontumor cell targets has been increasing. Ischemic and hypoxic stress is the most important condition for pathological and physiological angiogenesis [7]. Vascular endothelial cells have been observed to be the critical determinant in both the normal and tumoral tissue. In primary targets, the degree of radiation damage in the endothelial cells can significantly affect the treatment response [8]. Depending on tumor vasculature, phenotype, and microenvironment, it is different for normal tissue vasculature. The most important molecular difference of tumoral endothelial cells is their close relation with tumor-related cytokines [7,9,10]. VEGF and similar proteins released by the tumoral and normal tissues can send signals developing angiogenesis response by attaching to the tyrosine kinase receptor of the endothelial cell. Thus, the radiation resistance of tumoral endothelial cells can depend on tumor-related cytokines. Selective tumor vasculature targeting these cytokines can increase sensitivity to radiation [7,8,10].

It is suggested that the major determinant of radiotherapeutic response is tumor vasculature [11]. This relation shows that in addition to radiotherapy leading to direct cell death, the damage it causes to the vessels supplying the tumor leads to secondary cell death. As tumors need their vessels to survive, a small amount of damage in these vessels can lead to larger amounts of tumoral death. It is reported that tumors sensitive to radiotherapy have good vasculature, while tumors with little vasculature are more resistant to radiation [11]. In a study reported by Gorski et al., the way in which tumor vasculature affects radiotherapeutic response was evaluated, and it was stressed that radiotherapy increased VEGF release while VEGF was responsible for the resistance of endothelial cells to radiation [12]. This study showed that the tumor actively protects its vasculature from radiation damage. In certain other studies, similar results were obtained on the role of the combination of antiangiogenic agents and radiotherapy in improving endothelial cell sensitivity to radiation [13,14,16].

It is known that HIF-1α is activated in the event of hypoxia in normal tissues and solid tumors. Many studies have shown that, during hypoxia, HIF-1α is the main regulator of physiologic and pathologic angiogenesis signaling pathways. Normal tissue studies performed with radiation showed that as a result of HIF-1 increase, levels of VEGF, which is a positive influencing factor of the angiogenesis pathway, are also increased [17]. When the organism is exposed to factors like stressing radiation, heat, hypoxia, ischemia, and infection, stress granules that contain approximately half mRNA are activated. These granules change cell metabolism in the recovery of stress-related damage. eIF2 and TIA-1 act in change and adaptation. While formation of granules decreases eIF2 levels by eIF2 phosphorylation, TIA-1 levels are increased under the influence of stressing factors. TSP-1, which acts as an inhibitor endogenously on angiogenesis signaling pathways, is found at certain levels in normal tissue [18]. Higher levels of HIF-1 and VEGF, which increase with stressing factors in normal tissue studies, lead to TSP-1 down-regulation, while decreased VEGF leads to TSP-1 up-regulation. This is explained by the VEGF-mediated negative feedback mechanism.

Following exposure to radiation, VEGF, which acts on the angiogenesis signaling pathways, was increased. In the present study, all immunohistochemical evaluations revealed that VEGF staining was weak in Groups 1 (control) and 4 (euthanasia on day 7), medium in Group 2 (euthanasia after 24 h), and strong in Group 3 (euthanasia after 48 h). These findings show that at the vessel endothelium of tissues exposed to radiation, VEGF levels are increased slightly in vascular endothelial cells exposed to stress 24 h after radiation and strongly at 48 h. This increase was thought to be related to the strong increase of HIF-1α at 48 h. In the study by Rabbani et al., VEGF levels increased with the increase in HIF-1α [17]. The release of VEGF is an important process in angiogenesis signaling pathways and ionizing radiation increases VEGF release by increasing HIF-1α [12,17]. However, we determined that levels of HIF-1α and VEGF had regressed to control group levels at day 7. We administered a single fraction of 10 Gy of ionizing radiation. This may be the reason for the regression observed in HIF-1α and VEGF levels back to the control group levels at day 7. Imaizumi et al. in 2010 performed an animal study on normal tissues and revealed results similar to ours [19]. They exposed healthy mice to 1 fraction of 8 Gy, 15 Gy, and 20 Gy total body irradiation and observed that the VEGF levels of aortic endothelial cells decreased in comparison to the control group after 5 days. They concluded that high doses of ionizing radiation stopped endothelial cell proliferation, migration, and budding. It was indicated that in the tumoral environment, endothelial cells are not silent; they continue to multiply and are related to the microenvironment. However, it was also stated that, with radiation exposure, de novo angiogenesis was blocked, but the recurrence in irradiated areas being more resistant than the recurrence in nonirradiated areas could be because of the radiation-related inhibition of de novo angiogenesis. Our results are in accordance with this study. In our study, we observed that following radiation exposure, HIF-1α and VEGF, which are the positive influencing factors of the angiogenesis pathway, were increased immunohistochemically at 48 h and had regressed to the control group levels on day 7. In the study by Imaizumi et al., HIF-1 levels influenced by the hypoxic stress that could depend on radiation were not measured. In our study, after a single fraction of 10 Gy of radiation exposure, the increase in HIF-1α and VEGF at 48 h was determined immunohistochemically. Similarly, in the study by Rabbani et al., VEGF levels increased following the increase in HIF-1 levels [17]. This shows that after normal tissue radiation exposure, the positive influencing factors (HIF-1, VEGF) of the angiogenesis pathway are increased. VEGF increases the radiation resistance of the endothelial cells. Dicker et al. stated that the negative influence of this response can be eliminated with antiangiogenic medications and in their study performed on normal animals, along with radiation exposure, they used Cox-2 inhibitors that have a positive influence on the VEGF level of the angiogenesis pathway and succeeded in increasing apoptosis while inhibiting proliferation and migration [20].

It was reported that HIF-1 levels were regulated by the oxygen concentration, and following radiation treatment, tumor oxygenation changes with HIF-1 up-regulation [7,21]. Radiotherapy-induced HIF-1 activation is also related to reoxygenation [21]. In our study, among preparations stained with the HIF-1α antibody, Group 3 showed strong staining, while the degree of staining in other groups was significantly less. HIF-1α levels reaching their highest at 48 h showed that the hypoxic state, which depends on radiation, can be regulated with the increase in HIF-1α. Nevertheless, it is not correct to relate this condition to apoptosis. In tumoral tissues, the apoptosis in endothelial cells peaks at 4-8 h following radiation and the vascular damage typically starts after 48 h. However, the rate of endothelial cells entering apoptosis is between 0% and 8% following clinical doses of radiotherapy [13]. Li et al. performed a radiation-related apoptosis study on the vascular endothelial cells of nontumoral tongue tissue in rats and observed that the rates of apoptosis on days 5, 8, 14, 21, and 28 were 78.3%, 89.3%, 83.5%, 69.3%, and 47.3%, respectively [22]. Their findings support the finding that apoptosis does not happen at the same time in tumoral and nontumoral environments. Although apoptosis is stimulated by radiation in general, the main death mechanism may not be apoptosis in many cell types [23]. In our study, a single fraction of 10 Gy of radiation was given and the staining in HIF-1α was strong at 48 h; if fractioned radiation were used, different results could be obtained.

The RNA granules that emerge in mammalian cells exposed to heat, oxidation, radiation, and hypoxia are called stress granules. As a response to stress, the mammalian cell mRNA changes its cell metabolism for repairing stress-related damage. During this change and adaptation, RNA-binding TIA-1 also plays a role in the relation between eIF2 and stress granules [7,24,25,26]. When stress occurs once in the cell, the granules are depolarized. Kedersha and Anderson defined this in 2002 [26]. Stress granules can emerge in tissues exposed to stress a few hours before apoptosis. Stress granules can regulate protein translation in neurons during ischemia. Stress granules do not develop in environments in vitro because response to stress can only emerge in the microenvironment in vivo [7,26]. In our study, the eIF2 antibody showed strong immunohistochemical staining in the control group and Group 4, while weak staining was observed in Groups 2 and 3. Following irradiation of bladder vessel endothelium, the eIF2 level was lower than that of the control group at 24 h, then showed the tendency to increase at 48 h and reached the level of the control group on day 7. Stress granules can emerge in 15-30 min after the organism is exposed to stress. The eIF2 level decreases following its phosphorylation. The decrease in eIF2 levels in Group 2 confirms this. In tissues exposed to stress, stress granules emerge a few hours before apoptosis. During this change and adaptation, RNA-binding TIA-1 plays a role in the relation between eIF2 and stress granules [7]. The increasing tendency of eIF2 levels at 48 h and on day 7 can indicate the start of apoptosis and/or the cells’ recovery from stress [7,24,25,26]. This is supported in the study of Li et al. They determined apoptosis on day 5 [22]. eIF2 staining could have increased at 48 h and on day 7 because of apoptosis and/or the cells’ recovery from stress. However, in our study, apoptosis could not be determined; only the factors that increase and decrease before apoptosis were histochemically observed. Our method was not convenient for determining apoptosis.

In our study, the immunohistochemical evaluation performed with the TIA-1 antibody revealed that the control group was weakly stained, while Groups 2 and 4 showed medium staining and Group 3 was very strongly stained. This showed that no stressing factor existed in the control group, while in Groups 2 and 3, TIA-1 levels started to increase depending on stressing factors and reached a maximum in Group 3. 

TSP-1 is our only variable with a negative influence on angiogenesis signaling pathways. Ischemic or hypoxic stress is the most important condition for pathological or physiological angiogenesis [8]. Although many factors positively control the pathological and physiological pathways of angiogenesis, the most important endogenous regulator in down-regulation is TSP-1. TSP-1 prevents formation of vessel lumen [18,27]. In our study, immunohistochemical staining with the TSP-1 antibody revealed that the control group was very strongly stained, while staining in the other groups was very weak. However, the mean TSP-1 level at day 7 showed the tendency to increase when compared to the mean levels at 24 and 48 h. This can be explained as follows: in our study, at the angiogenesis pathway that was the result of radiation-related hypoxia, the positive influencing factor HIF-1α, which is stressing, showed strong staining in Group 3 while staining weakly in Group 4. eIF2, which is the determinant of stress granules, showed weak staining in Groups 2 and 3, while getting close to control group levels in Group 4. Under the influence of the stressing factor, TIA-1 showed medium staining in Group 2 and strong staining in Group 3, while regressing to the control group levels in Group 4. VEGF showed strong staining in Group 3. Increased levels of positive influencing factors can explain TSP-1, which acts as an endogenous inhibitor at the angiogenesis pathway, being weakly stained in Groups 2 and 3, while the slight increase in staining in Group 4 can be explained as the decrease in positively influencing factors of the angiogenesis pathway leading to the increased immunohistochemical staining of TSP-1. At the endothelial cells of the irradiated vessel, the hypoxic stress can lead to the release of HIF-1 or indirect HIF-1 increase by the increase in ROS, and VEGF release [7]. The positively influencing factors (HIF-1α, VEGF, eIF2, and TIA-1) of the angiogenesis pathway could be stimulated and this might have decreased TSP-1, which is acting as an endogenous angiogenesis inhibitor. Mean HIF-1α and VEGF levels increased at 24 and 48 h because of stressing factors and decreased after 7 days, and this can explain the TSP-1 levels reaching the control group levels after 7 days. Suzuma et al. reported that increased VEGF leads to TSP-1 down-regulation, while decreased VEGF leads to TSP-1 up-regulation. This can be explained by a VEGF-mediated negative feedback mechanism [28]. 

## CONCLUSION

Serum levels of angiogenic cytokines decrease after radiotherapy in patients with hematologic malignancy and become undetectable in patients with complete remission [29]. However, by exposing normal tissue to radiation, we determined that the positively influencing factors of the angiogenesis pathway increased, while the negatively influencing factor decreased. Radiation can start physiological angiogenesis in normal tissues and can accelerate the healing of damaged normal tissue. The normal and tumoral tissues respond to radiation differently. In the future, molecular studies designed with fractioned doses and diagnostic angiogenesis studies will lead to a better understanding of this subject.

## CONFLICT OF INTEREST STATEMENT

None of the authors of this work have any conflicts of interest, including specific financial interests, relationships, and/or affiliations, relevant to the subject matter or materials included. 

## Figures and Tables

**Table 1:**
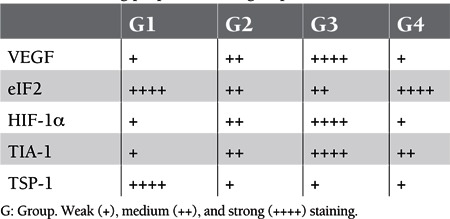
Staining properties of all groups
